# Integrative Multi-Omics Identify Key Secondary Metabolites Linked to Acid Tolerance in *Leptospirillum ferriphilum*

**DOI:** 10.3390/microorganisms13112493

**Published:** 2025-10-30

**Authors:** Yiran Li, Jiejie Yang, Xian Zhang, Luhua Jiang, Shiqi Chen, Manjun Miao, Yili Liang, Xueduan Liu

**Affiliations:** 1 School of Minerals Processing and Bioengineering, Central South University, Changsha 410083, China; l18163747285@163.com (Y.L.); jiejieyang212@foxmail.com (J.Y.); jiangluhua@csu.edu.cn (L.J.); 235611016@csu.edu.cn (S.C.); 235612083@csu.edu.cn (M.M.); 2 Key Laboratory of Biohydrometallurgy, Ministry of Education, Changsha 410083, China; 3 Department of Occupational and Environmental Health, Xiangya School of Public Health, Central South University, Changsha 410031, China; zixuange2010@126.com

**Keywords:** *Leptospirillum ferriphilum*, secondary metabolites, biosynthetic gene cluster, quorum sensing, environmental adaptation

## Abstract

Acid mine drainage (AMD) environments feature extreme acidity (pH ≤ 2) and high heavy metal concentrations. Acidophiles survive these conditions through unique genetic adaptations and secondary metabolite (SM) pathways. *Leptospirillum ferriphilum*, known for its acid and heavy metal resistance, serves as a model for AMD bioremediation, though systematic multi-omics studies on its key SMs and biosynthesis pathways remain underexplored. In this study, *L. ferriphilum* YR01 was isolated and identified from the AMD of the Zijinshan copper mine, China. Pangenomic analysis revealed that YR01 possesses the largest number of genes (2623) among the eight sequenced *L. ferriphilum* strains. Comparative genomics, antiSMASH, BiG-SCAPE, and metabolomic analyses (LC-MS and HPLC-MS) were integrated to comprehensively explore its biosynthetic capacity. A total of 39 biosynthetic gene clusters (BGCs) were identified, of which 60% shared <50% similarity with known clusters, indicating substantial novel biosynthetic potential. The sequence alignment of SM biosynthetic gene clusters (BGCs) demonstrated the potential of *L. ferriphilum* to synthesize conserved clusters for ectoine, choline, carotenoids, terpenoids, and terpene precursors. YR01 harbors complete BGCs for all five SM types. Notably, key nonribosomal peptide synthetase (NRPS) modules implicated in *N*-acyl homoserine lactone (AHL) synthesis were identified. Untargeted metabolomics (LC-MS) revealed the production of diverse SMs (18 types) putatively involved in environmental adaptation, including phosphocholine, carotenoids (e.g., anteraxanthin), cholera autoinducer-1 (CAI-1), and multiple AHLs. Targeted detection (HPLC-MS) further confirmed that YR01 could produce ectoine (0.10 ng/mL) and specific AHLs (C_14_-HSL, C_12_-HSL, C_12_-OH-HSL), which were beneficial for the survival of the strain in extremely acidic environments and interspecies communication through SMs. This study represents the first comprehensive multi-omics characterization of BGCs in *L. ferriphilum* and experimentally validates the production of key SMs. Collectively, this study provides a comprehensive elucidation of the SM biosynthetic repertoire and environmental adaptation strategies in *L. ferriphilum*, advancing our understanding of microbial adaptation and interspecies communication in AMD systems, and offering potential implications for biomining applications.

## 1. Introduction

Acid mine drainage (AMD) ecosystems represent globally distributed extreme acidic environments [1]. AMD is characterized by extreme acidity, high concentrations of heavy metals, and elevated sulfate levels, posing serious threats to both the ecological environment and human health. Acidophiles naturally accumulate in AMD environments and play a key role in bioremediation and biomining. Acidophiles inhabiting these environments have evolved unique metabolic pathways and adaptation mechanisms [2]. *Leptospirillum ferriphilum*, a dominant species in AMD, maintains activity under low pH, high temperature, and high salinity conditions, demonstrating multiple environmental adaptations [3]. Functioning as a key chemolithoautotrophic iron-oxidizing bacterium in acidic environments, *L. ferriphilum* derives energy from the oxidation of ferrous ions to ferric ions, concomitant with sulfide release [3]. Its efficient ferrous oxidation ability makes it a core microorganism for biomining, particularly in the biomining of metals such as copper and iron [4]. During the AMD biofilm formation, *L. ferriphilum* often acts as a pioneer colonizer, stabilizing mineral surfaces by forming biofilms and providing a critical microhabitat for subsequent microbial communities [5]. Given its strong environmental adaptability and iron-oxidizing metabolism, *L. ferriphilum* plays a key role in biomining and environmental remediation [6]. Moreover, the study of quorum sensing (QS) in acidophiles, including DSF and AHL signaling molecules, has positioned *L. ferriphilum* as an emerging model for microbial signaling studies in extreme environments. Previous studies have shown that *Leptospirillum* and other acidophiles employ multiple metabolic strategies—such as osmoprotectant production, QS regulation, and metal ion resistance—to survive under low-pH and high-metal conditions [7,8,9]. These adaptive mechanisms are largely driven by specific secondary metabolic pathways encoded in their genomes.

Secondary metabolites (SMs) are essential non-growth-associated compounds synthesized by microorganisms during specific growth phases [7]. In extreme acidic environments, these metabolites play crucial roles in microbial interspecies interactions, adaptation to environmental stresses, and potential environmental remediation [8]. Evidence indicates that *Leptospirillum* spp. produce diffusible signal factor (DSF) QS signaling molecules [9]. For instance, during pyrite colonization, *L. ferriphilum*, *L. ferrooxidans*, and other *Leptospirillum* strains produce DSF analogs that inhibit competing iron oxidizers, thereby sustaining their ecological competitive advantage [9,10]. Notably, the expression of QS-related genes in *Leptospirillum* is tightly regulated by environmental pH and metal ion concentrations [10]. Furthermore, high osmolarity, induced by elevated salinity and sulfate concentrations, triggers ectoine (1,4,5,6-tetrahydro-2-methyl-4-pyrimidinecarboxylic acid) biosynthesis in *Leptospirillum* [11,12]. Ectoine accumulates intracellularly, functioning as a key osmoprotectant by maintaining cellular structural integrity and preventing protein denaturation [13,14]. While ectoine enhances acidophile adaptability and QS molecules facilitate ecological competitiveness, comprehensive SM synthesis capabilities are not ubiquitous among all species.

SM biosynthesis is primarily governed by biosynthetic gene clusters (BGCs), which are typically clustered within the genome [15]. Investigating the BGC potential of *L. ferriphilum* is particularly relevant, considering that approximately 92% of PKS/NRPS pathways in AMD remain uncharacterized, representing a largely untapped reservoir of bioactive compounds [15]. Among the AMD-associated microorganisms, terpene BGCs predominate, with ribosomally synthesized and post-translationally modified peptides (RiPPs) and NRPS BGCs also prevalent; however, the functions and products of many clusters remain elusive [16]. QS mediated by AHL signaling molecules constitutes a crucial regulating mechanism in biomining. Although *N*-acyl homoserine lactone (AHL) production has not been directly detected in *Leptospirillum*, genomic analysis revealed that Group II strains carry typical LuxI/R QS system elements [3,10,17]. The *luxI* gene encodes AHL synthases, while *luxR* encodes AHL-dependent transcription factors that regulate biofilm formation and intraspecies communication [18]. *Leptospirillum* Group II synthesizes ectoine and hydroxyectoine via the *ectABCD* gene cluster and a specific transporter, and Group III strains lack functional ectoine synthesis genes and transporters, potentially relying on exogenous uptake [11]. To efficiently identify and compare these BGCs, we employed an integrated bioinformatics pipeline combining antiSMASH and BiG-SCAPE, which allows prediction of biosynthetic functions and assessment of BGC modularity. Genome mining, utilizing sequencing combined with bioinformatics tools (e.g., antiSMASH), represents an effective strategy for identifying the SM biosynthetic potential of extremophiles [19]. This approach can unveil pathways for specialized metabolites silent under standard laboratory conditions and holds promise for enabling large-scale compound production via heterologous expression. Currently, the genomic studies of *L. ferriphilum* have focused on its basic metabolic pathways (e.g., iron oxidation, nitrogen fixation), while systematic investigation and functional characterization of its sm-BGCs remain limited [19].

In this study, stain YR01 was isolated from the traditional AMD of the Zijinshan copper mine, China. The objective of this study was to identify, compare, and validate key secondary metabolites in *L. ferriphilum* through an integrated multi-omics approach combining genomics, metabolomics, and bioinformatics. Taxonomic identification and characteristic analysis were conducted using methods such as 16S rRNA, whole-genome sequencing, and ANI, confirming its classification as the species *L. ferriphilum*. Genomic research focusing on sm-BGCs showed that *L. ferriphilum* possesses conserved domain gene clusters for the synthesis of various SMs. Untargeted (LC-MS) metabolomics analysis indicated that YR01 could produce a variety of SMs beneficial for the environmental adaptation. Finally, targeted detection demonstrated that YR01 produced ectoine and various AHLs by HPLC-MS. This study represents the first comprehensive multi-omics characterization of sm-BGCs in *L. ferriphilum*, providing insights into its environmental adaptation mechanisms and microbial interactions in AMD systems.

## 2. Materials and Methods

### 2.1. Cultivation, DNA Extraction, and Sequencing of Strain YR01

In this study, strain YR01 was isolated from AMD samples collected on 22 August 2023, from the Zijinshan copper mine (25°10′41″ N–25°11′44″ N, 116°24′00″ E–116°25′22” E) in Fujian Province, China. The isolation process involved inoculating 10.0 mL aliquots of liquid samples into modified 9K medium (pH 2.0) supplemented with 44.7 g/L FeSO_4_·7H_2_O. Cultivation was conducted at 40 °C under aerobic conditions using a rotary shaker at 180 rpm. The base 9K medium composition consisted of the following (per liter): (NH_4_)_2_SO_4_ (3.00 g/L), K_2_HPO_4_ (0.50 g/L), KCl (0.10 g/L), Ca(NO_3_)_2_ (0.01 g/L), and MgSO_4_·7H_2_O (0.50 g/L). Following primary enrichment, strain YR01 was purified through serial gradient dilution techniques and subsequently subjected to genome sequencing. Strain YR01 cultured to the exponential stage was centrifuged at 12,000× *g* and 4 °C for 10 min, and then DNA extraction was performed using the QIAamp DNA Mini Kit (Qiagen, Hilden, Germany). The fluorescence quantification of the extracted DNA was performed using the TBS-380 instrument (Turner BioSystems, Sunnyvale, CA, USA). DNA samples with OD260/280 ≥ 1.8–2.0 and content > 10 µg were selected for sequencing. Subsequently, hybridization sequencing was performed using the third-generation PacBio RS and the second-generation Illumina PE150 platform (Shanghai Majorbio bio—pharmaceuticaltechnology Co., Shanghai, China) [20]. The offline data were preprocessed using the software fastp v0.23.2 to obtain clean data, and then, the sequence was assembled using the three pieces of software SOAP denovo v2.04, SPAdes v3.15.5 and Abyss v2.3.4. The assembly results of the three software were compared, and the optimal kmer was selected, that is, the assembly result with the least scaffold value. Subsequently, the software GapClose v1.2.1 was used to fill the holes in the initial assembly results and filter out the fragments that were <500 bp. The screened fragments were used for subsequent evaluation and analysis and gene prediction.

### 2.2. Genomics Analysis of Strain YR01

The genomic datasets were primarily retrieved from National Center for Biotechnology Information (NCBI) [21], supplemented by one novel strain isolated and screened in this study, with a data cutoff date of 1 March 2025. A phylogenetic tree of YR01 was constructed using the neighbor-joining method based on the 16S rRNA sequences in MEGA v12 [22]. The whole-genome sequences of *L. ferriphilum* isolates from acidic mine drainage environments, available in public databases, were selectively used as the main research objects. The phylogenetic relationships among different *L. ferriphilum* strains were then evaluated using an alignment-free composition vector method implemented in CVTree4 [23]. Final tree visualization and annotation were performed using the web-based Chiplot platform [24], ensuring robust phylogenetic resolution. Orthologous Average Nucleotide Identity (OrthoANI) values between these strains were calculated and visualized as heatmaps through the OAT: OrthoANI toolkit (v0.93.1) [25]. Pangenome analysis was conducted using the Bacterial Pan Genome Analysis tool (BPGA v1.3) with the integrated USEARCH program (v11.0.667) [26]. A sequence identity threshold of 50% was applied for clustering homologous genes.

### 2.3. Identification, Cluster Similarity Analysis, and Evolutionary Analysis of BGCs

As shown in Table 1, all whole-genome-sequenced strains (8 strains) of *L. ferriphilum* were retrieved from the NCBI. Secondary metabolite BGCs were predicted and annotated locally using the antiSMASH 8.0 database [19]. AntiSMASH outputs provided comprehensive BGC profiles including cluster type, genomic coordinates, most similar known cluster, and chemical similarity percentage. Resultant data were standardized and visualized using the software Origin 2024.

AntiSMASH-generated GenBank files served as the input for Biosynthetic Gene Cluster Similarity Analysis and Prediction Engine (BiG-SCAPE) [27]. The analytical pipeline executed the following: BGC-encoded sequences were annotated via HMMER’s hmmerscan against the Pfam database, converting each BGC into a linear string of Pfam accessions ordered by genomic position. BiG-SCAPE categorized antiSMASH-predicted products into eight classes per antiSMASH rule: Saccharides, NRPS, Others, Terpene, PKS I, PKS other, RiPPs, and PKS/NRPS Hybrids. BiG-SCAPE allows users to customize parameters (e.g., --clan_cutoff and --cutoff) to balance clustering precision and breadth. The similarity cutoff (--cutoff) was set to 0.3, and the clan cutoff (--clan_cutoff) was set to the default value. “Relax” thresholds (0.5) are suitable for grouping BGCs of the same compound [28]. For comparative analysis, the functional diversity of BGCs across strains was visualized using Chiplot to generate a structured representation of their metabolic potential.

### 2.4. Untargeted Metabolomics Using LC-MS

*Leptospirillum ferriphilum* YR01 was cultivated in modified 9K medium (composition identical to Section 2.1) supplemented with 60 g/L FeSO_4_·7H_2_O. Cultures were maintained at pH 2.0 and 38 °C with 180 rpm agitation for 72 h [29]. Samples were fractionated into intracellular and extracellular components. Each fraction underwent triple extraction with 1000 µL ice-cold extraction solvent (methanol/acetonitrile = 1:1 *v*/*v*, containing 20 mg/L internal standard). Samples were subjected to bead-beating (45 Hz, 10 min) and probe sonication (20 min, ice-water bath). Phase separation was achieved with centrifugation (12,000 rpm, 15 min, 4 °C). Solvent evaporation was performed using a vacuum concentrator. Dried metabolites were reconstituted in 160 µL solvent (acetonitrile/water = 1:1 *v*/*v*). Reconstituted samples were vortexed (30 s) and sonicated (10 min, ice-water bath). Clarification was performed using centrifugation (12,000 rpm, 15 min, 4 °C). A 120 µL volume of supernatant was transferred to 2 mL amber vials, with 10 µL aliquots pooled for Quality Control (QC) samples [30,31]. Metabolite profiling was conducted using a Waters Acquity I-Class PLUS UPLC system coupled with a Waters Xevo G2-XS QTOF mass spectrometer. Separation employed an Acquity UPLC HSS T3 column (1.8 µm, 2.1 × 100 mm) with a 2 µL injection volume. Mass range: *m*/*z* 50–1200 [32]. Raw data acquired via MassLynx V4.2 underwent peak picking, alignment, and feature detection using the software Progenesis QI v2.0. Metabolite annotation was performed against online METLIN database, public metabolite repositories, and custom spectral libraries with concomitant theoretical fragmentation pattern matching. Resultant data were standardized and visualized using the software Chiplot accessed on 13 May 2025 (https://www.chiplot.online/) and Origin 2024. For LC-MS and HPLC-MS analyses, each culture was prepared in biological triplicate (*n* = 3) to ensure statistical robustness.

### 2.5. Identification of Key Secondary Metabolites Using HPLC-MS

*L. ferriphilum* YR01 was cultivated in modified 9K medium (composition identical to Section 2.4). Centrifugation was performed at 4000× *g* for 2 min, followed by supernatant filtration. Cellular disruption: 200 mL fermentation broth was subjected to probe sonication for a total duration of 30 min to ensure complete cell lysis and the release of intracellular target compounds. Primary extraction: the lysate was mixed with ethyl acetate in a 1:1 volumetric ratio, vigorously agitated, and incubated overnight (18 h) for phase separation. Organic-phase concentration: Rotary evaporation was performed at 65 °C in a water bath. Post-evaporation, the residual extract was reconstituted by dual rinsing with 2 mL methanol, with eluates pooled into a 5 mL centrifuge tube. Filtrate preparation: the concentrate was filtered through a 0.22 μm organic-phase-compatible membrane into HPLC vials [33].

**Ectoine Quantification Protocol:** For ectoine quantification, CAS 96702-03-3 (97%, MDL: MFCD03419286) was used as the internal standard. Calibration curves were prepared using serial dilutions of the internal standard to determine the linear range and limit of detection, enabling precise quantification of ectoine in the samples. An ectoine standard stock solution (500 µg/L) was prepared in methanol. Working standards were serially diluted in methanol to generate a 500 ng/L calibration solution. The chromatographic conditions are shown below. Column: Poroshell 120 HILIC (2.7 µm, 2.1 × 150 mm). Mobile phase: A (0.1% formic acid in ultrapure water), B (0.1% formic acid in acetonitrile), and isocratic elution (A:B = 40:60 *v*/*v*). Parameters: flow rate (0.30 mL/min), column temperature (30 °C), injection volume (10 µL), and quantitative ion (*m*/*z* 143.1 [M + H]^+^) [34,35].

**AHL Quantification Protocol:** For AHLs, internal standards were purchased from Sigma-Aldrich (St. Louis, MO, USA) (>98% purity, analytical grade), and calibration curves were similarly prepared to ensure precise and reliable quantification. Individual stock standard solutions of each of the five signal molecules were prepared in methanol at a concentration of 500 µg/L. Aliquots of 100 µL from each stock solution were combined and diluted with methanol to prepare a mixed standard solution. This mixed standard solution was then serially diluted with methanol as required to achieve a final concentration of 500 ng/L for the working mixed standard solution. Data acquisition was performed primarily using HPLC-MS. Specific MRM ion transitions were monitored and recorded based on the elution time windows of the respective metabolites [36]. Resultant data were standardized and visualized using the software Origin.

## 3. Results and Discussion

### 3.1. Taxonomic Identification and Characterization of Strain YR01

**Figure 1:**
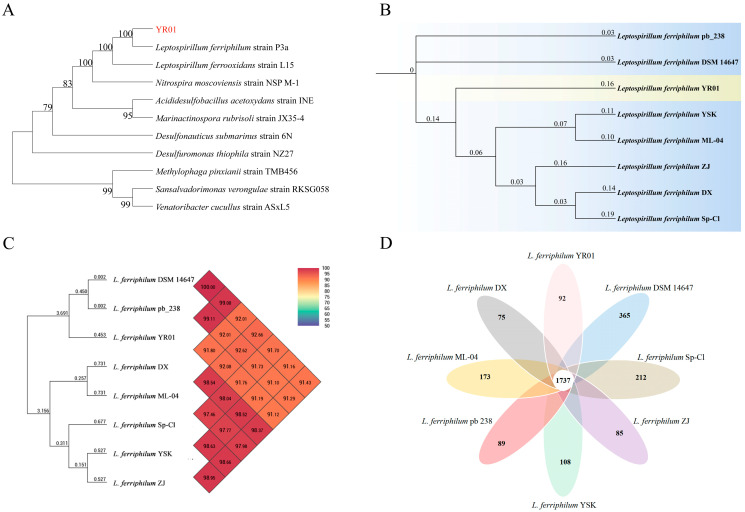
Genomic characteristics and homology analysis of YR01. (**A**) Phylogenetic tree based on 16S rRNA gene. (**B**) Whole-genome-based phylogenetic tree of *L. ferriphilum* strains with neighboring standard species. (**C**) Heatmap of OrthoANI values between *L. ferriphilum* YR01 and known *L. ferriphilum* strains. (**D**) Pangenome analysis of *L. ferriphilum* YR01 and known *L. ferriphilum* strains.

To elucidate the evolutionary relationship between strain YR01 and related species, a phylogenetic tree was constructed based on 16S rRNA gene sequences from nine strains. As shown in Figure 1A, YR01 was most closely related to *L. ferriphilum* strain P3a (99.79%). The genome size of strain YR01 was 2,390,881 bp, with a total GC content of 54.41%. The genome encodes 2623 protein-coding genes, 2 sRNA genes, and 54 tRNA genes. Multiple strains of the species *L. ferriphilum* have been isolated from different sources (Table 1). To further explore evolutionary relationships, a whole-genome-based phylogenetic tree was constructed using eight *L. ferriphilum* model strains (Figure 1B). Conventionally, average nucleotide identity (ANI) values exceeding 95% indicate strains belonging to the same recognized microbial species [37]. ANI values between YR01 and some of the reference *L. ferriphilum* strains exceeded this threshold, confirming their close phylogenetic affiliation. Phylogenetic analysis positioned YR01 closest to *L. ferriphilum* pb_238 and *L. ferriphilum* DSM 14647 (Figure 1C). Collectively, whole-genome sequencing, ANI values, and phylogenetic analyses confirm the classification of strain YR01 as *L. ferriphilum*. Consequently, YR01 was designated as *Leptospirillum ferriphilum* YR01.

**Table 1:** Genomic characteristics of analyzed *L. ferriphilum*.

Strain	Size (Mb)	Level	GC%	Contig	Gene	Isolation Source	Reference
*Leptospirillum ferriphilum* pb_238	2.611	Contig	54.0	2	2603	Acid mine drainage	/
*Leptospirillum ferriphilum* ML-04	2.406	Complete	54.5	1	2394	Acidic water (Yunnan, China)	[38]
*Leptospirillum ferriphilum* YSK	2.331	Complete	54.5	1	2284	Acid mine drainage (Jiangxi, China)	[29]
*Leptospirillum ferriphilum* DSM 14647	2.406	Contig	54.0	18	2383	Enrichment culture (Peru)	[39]
*Leptospirillum ferriphilum* DX	2.361	Contig	54.5	30	2328	Acid mine drainage (Jiangxi, China)	[40]
*Leptospirillum ferriphilum* Sp-Cl	2.476	Contig	54.5	74	2468	Industrial bioleaching solution (Chile)	[41]
*Leptospirillum ferriphilum* ZJ	2.341	Contig	54.5	104	2345	Acid mine drainage (Fujian, China)	[40]
*Leptospirillum ferriphilum* YR01	2.350	Scaffold	54.4	12	2623	Acid mine drainage (Fujian, China)	This study

The pangenome analysis of the eight *L. ferriphilum* strains delineated three distinct genomic components: the core genome, accessory genome, and unique genome [42]. This analysis identified 1737 core genes shared among all strains. The genome of *L. ferriphilum* YR01 contained a relatively large number of genes (2623), including 92 unique genes (Figure 1D). Further analysis revealed that *L. ferriphilum* DSM 14647 has the greatest number of unique genes (365).

### 3.2. Identification and Clusters of BGCs

**Figure 2:**
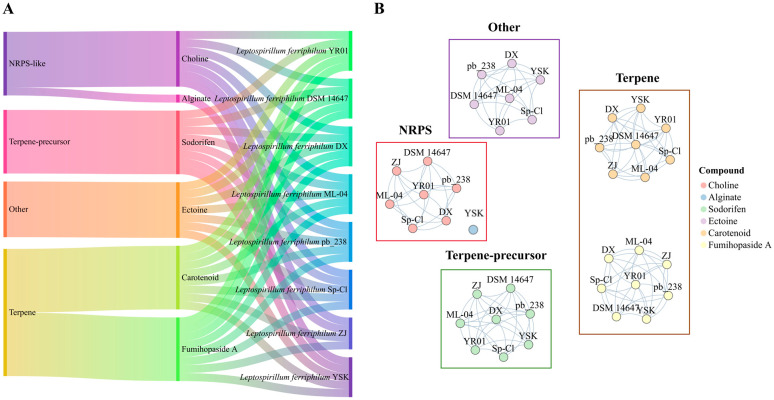
Classification and clustering of BGCs in *L. ferriphilum*. (**A**) Classification of BGCs in *L. ferriphilum*. (**B**) Clustering diagram of BGCs in *L. ferriphilum*.

Utilizing the antiSMASH, 39 secondary metabolite BGCs were successfully annotated across the genomes of eight *L. ferriphilum* strains (Figure 2A). Each *L. ferriphilum* strain harbored four to five BGCs, with an average of five BGCs per strain. Terpene-related BGCs (encompassing terpene and terpene-precursor types) predominated, with an average of three clusters per strain. Each strain possessed one NRPS-BGC and one Other-BGC. Notably, *L. ferriphilum* ZJ was the only one lacking the Other-BGC. The Terpene-BGCs encode biosynthetic pathways for carotenoids and fumihopaside A [43,44]. The Terpene-precursor-BGCs are responsible for sodorifen biosynthesis [45]. The NRPS-BGCs encode pathways for choline and alginate production and possess domains associated with QS signal molecule receptors [46]. The Other-BGCs encode the biosynthesis of ectoine.

To identify orthologous clusters among BGCs in various strains, a similarity network was constructed based on BGC sequences. As shown in Figure 2B, the examination of BGC sequence similarity revealed that the connected components aligned with 39 gene cluster families (GCFs), comprising 8 GCFs related to NRPS, 16 GCFs associated with terpenes, 8 GCFs linked to terpene precursors, and 7 GCFs pertaining to Other-BGCs. Furthermore, sequence similarity analysis indicated that 60% of *L. ferriphilum* BGCs exhibited <50% similarity with the characterized clusters. This finding underscores that the majority of *L. ferriphilum* sm-BGCs are highly specific and distinct from known microbial clusters.

#### 3.2.1. Key Clusters of Ectoine

**Figure 3:**
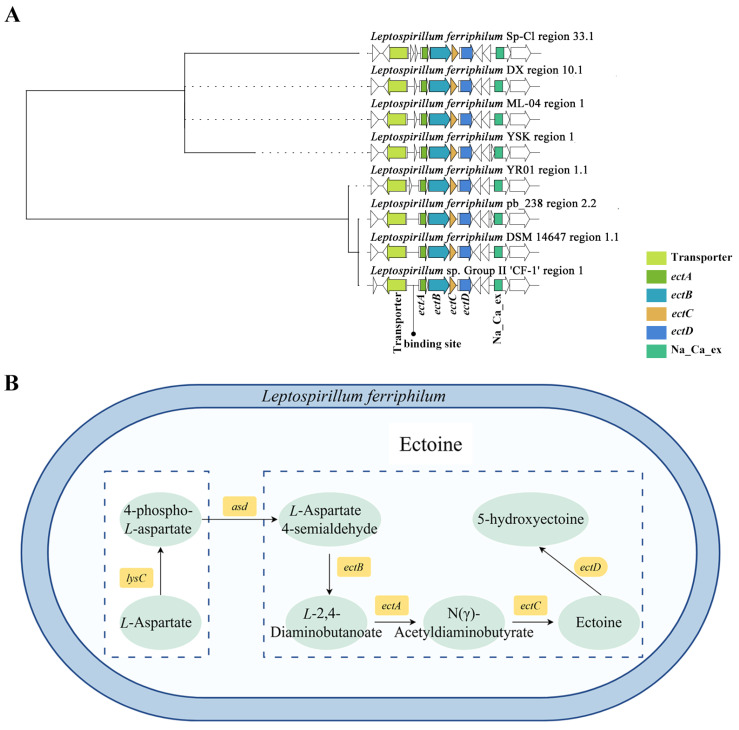
Biological synthesis of ectoine in *Leptospirillum*. (**A**) Clustering phylogenetic tree of synthetic gene cluster of ectoine in *Leptospirillum*. Colored modules represent genes with significant roles, while white modules represent genes with unknown functions. (**B**) The complete synthesis pathway of ectoine.

To thoroughly understand the relationship between “Other” BGC types and ectoine synthesis, sequence alignment analysis was performed on the key modules of this BGC. Phylogenetic tree construction based on clustering results revealed that the BGC of ectoine in *L. ferriphilum* was highly conserved (Figure 3). Beyond the core *ectABCD* genes, the cluster contains key auxiliary domains, including a binding site upstream of the ectoine synthase family, a specific transporter preceding the binding site, and a sodium/calcium exchanger protein. Notably, *L. ferriphilum* pb_238, *L. ferriphilum* DSM14647, and *Leptospirillum* sp. Group II CF-1 lacked an ORF with unknown function located at the front end of the BGC. *L. ferriphilum* Sp-Cl had two ORFs with unknown functions at the same position. This ORF was located between the transcription factor-binding site RutR (regulator of the pyrimidine and purine metabolism) and the transcription factor-binding site AfsR (pleiotropic regulatory for antibiotic production). Analysis revealed that, in some strains of *L. ferriphilum*, due to the deletion of the ORF in the promoter, only the binding sites for RutR and AfsR were retained. This may lead to a decrease in the binding ability of transcription factors (TetR), thereby affecting the expression of ectoine synthesis genes [47]. Ectoine accumulates intracellularly, functioning as a key osmoprotectant that maintains cellular structural integrity and prevents protein denaturation under environmental stresses such as high osmolarity and metal toxicity. While ectoine enhances the adaptability of acidophiles, variations in gene cluster organization may influence the regulation or metabolic flux of ectoine biosynthesis, warranting further experimental investigation. Furthermore, analysis indicated that *L. ferrooxidans* lacks a homologous ectoine synthesis cluster. Ectoine appear to be exclusively distributed in *L. ferriphilum* and an unidentified *Leptospirillum* sp. Previous studies identified genes encoding ProP proteins in *Leptospirillum* spp. genomes, potentially involved in the transmembrane transport of ectoine [48]. Additionally, a specific ectoine transporter gene is located upstream of the *ectABCD* operon in Group II clusters, whereas Group III clusters lack this gene [49].

Ectoine was the only secondary metabolite BGC among all *L. ferriphilum* with a sequence similarity exceeding 70%, and it was classified within the “Other” category. Among them, the sequence similarity of ectoine in strain YR01 exceeded 79%. The ectoine biosynthesis cluster in *L. ferriphilum* comprises three genes: *ectA*, *ectB*, and *ectC* [50,51]. The cluster for hydroxyectoine biosynthesis consists of four genes: *ectA*, *ectB*, *ectC*, and *ectD* [51,52]. The ectoine synthase family encompasses several synthase proteins. The *ectA* gene encodes diaminobutyric acid acetyltransferase. The *ectB* gene encodes diaminobutyrate-2-oxoglutarate aminotransferase (aminotransferase class-III). The *ectC* gene encodes ectoine synthase. The *ectD* gene encodes Phytanoyl-CoA Dioxygenase (PhyH). These proteins collectively constitute the complete ectoine biosynthetic pathway. Aspartic β-semialdehyde serves as the primary precursor for ectoine synthesis and is also the substrate for diaminobutyrate aminotransferase EctB. EctA catalyzes the conversion of EctB’s product, 2,4-diaminobutyric acid (DABA), into *N*-γ-acetyl-2,4-diaminobutyric acid (ADABA). Finally, synthase EctC cyclizes ADABA to generate ectoine.

#### 3.2.2. Key Clusters of NRPS

**Figure 4:**
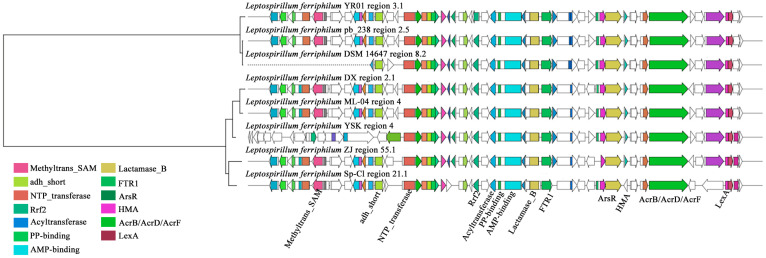
Clustering phylogenetic tree of synthetic gene cluster of NRPS in *L. ferriphilum*. Sequence alignment of AHL synthase gene cluster containing SAM and ACP modules in *L. ferriphilum*.

Within the GCF0028 gene cluster family of *L. ferriphilum*, the compound exhibiting the highest similarity to the sm-BGCs in this family was predicted to be choline, a NRPS (Type I) compound [53]. As an osmotic regulator, choline may enable microorganisms to metabolize pollutants (such as heavy metals) more efficiently by maintaining cell viability [54]. As illustrated in Figure 4, sequence alignment revealed high similarity among the core components within the *L. ferriphilum* NRPS-type BGCs. These include proximally positioned domains such as S-adenosylmethionine (SAM)-dependent methyltransferase, Short-Chain Dehydrogenase (SDR), and nucleotidyl transferase. Domains flanking key sites comprise iron-dependent transcriptional regulator (Rrf2 family), essential acyltransferase, phosphopantetheine attachment site (for 4′-phosphopantetheine prosthetic group activation), AMP-binding enzyme (characteristic of adenylation domains), metallo-beta-lactamase superfamily domain, and iron permease FTR1 family protein. Distally located domains include arsenical pump membrane protein (ArsR), heavy-metal-associated (HMA) domain, AcrB/AcrD/AcrF family integral membrane protein (associated with efflux), phosphoglucose isomerase, and LexA repressor DNA-binding domain. Notably, multiple heavy-metal-associated domains, such as Rrf2, iron permease, arsenical pump membrane protein, and HMA domain, were present near the ACP domain responsible for QS substrate specificity. *L. ferriphilum* YSK and *L. ferriphilum* DSM 14647 notably lack SAM-dependent methyltransferase and SDR, enzymes linked to QS signal molecule synthesis. Additionally, *L. ferriphilum* Sp-Cl was deficient in the heavy-metal-associated domain and phosphoglucose isomerase. Among these, the iron-dependent transcriptional regulator Rrf2, positioned near critical sites, may play a significant role in activating the substrate-specific ACP domain involved in AHL QS signal molecule synthesis [55].

NRPS-type secondary metabolite BGCs are often closely associated with molecules such as QS signal molecules, which play a crucial regulatory role in interspecies relationships within this acidophilic microbial system [56]. A complete NRPS/PKS module acyl carrier protein (ACP) domain was predicted within the NRPS-type BGCs present in the genome of each *L. ferriphilum* strain. Catalyzed by AHL synthase, acyl-ACP derived from the fatty acid metabolic pathway serves as a substrate alongside SAM to synthesize AHL-type QS signal molecules [57]. The ACP acquires an acyl chain from the fatty acid metabolism pathway and transfers it to the amino group of SAM. Subsequent lactonization then forms the AHL molecule [47]. The length and modification of the acyl chain carried by different ACPs directly determine the structure of the resulting AHL [58]. Consequently, the acyl chain specificity of the ACP is a key factor governing the AHL molecular diversity. The synthetic capability and pathway for different AHL types depend not only on the specific acyl-ACP substrate types available within each microorganism but also on the structural specificity of each AHL synthase enzyme [59]. The study suggests that, while all eight strains might synthesize choline, only *L. ferriphilum* YR01, pb_238, and ML-04 might produce AHL-type QS signal molecules.

#### 3.2.3. Key Clusters of Terpenoid

**Figure 5:**
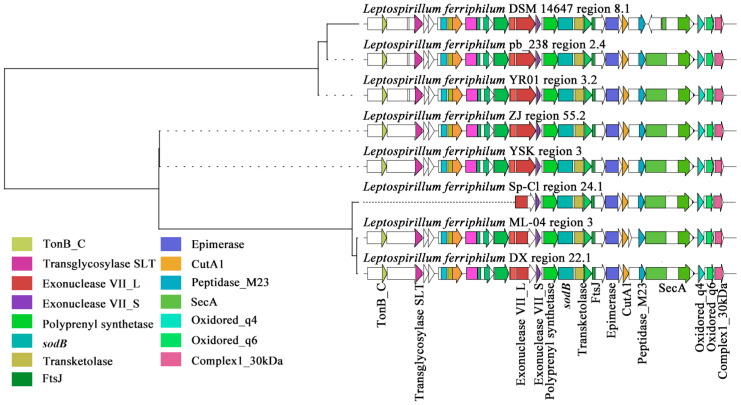
Clustering phylogenetic tree of synthetic gene cluster of *L. ferriphilum* terpene precursors.

The terpenoid BGCs of *L. ferriphilum* mainly cluster into three GCFs. Two families are of terpenoids, and one family is of terpenoid precursors. Within the GCF0031 gene cluster family of *L. ferriphilum*, the compound that exhibited the highest similarity to the secondary metabolite BGCs in this family was predicted to be sodorifen, a precursor of terpenoid-type compounds (Figure 5). Sodorifen is a naturally occurring volatile organic compound (VOC) characterized by an unusual polymethylated bicyclic hydrocarbon structure (C_16_H_26_) [45]. Studies on *Serratia plymuthica* revealed that its sodorifen-encoding BGC comprises four genes, two of which (*sodA*, *sodB*) are homologs of genes encoding enzymes of the non-mevalonate pathway, postulated to enhance the availability of the sodorifen precursor farnesyl pyrophosphate (FPP) [60]. A previous analysis of *S. plymuthica* determined that only two enzymes were required to produce sodorifen starting from FPP: an SAM-dependent methyltransferase with additional cyclization activity (SodC) and a terpene cyclase (SodD) [61]. Through sequence alignment and evolutionary analysis, this study revealed that, in contrast to *S. plymuthica*, the key enzymes for sodorifen biosynthesis in *L. ferriphilum* were likely a distinct SAM-dependent methyltransferase (FtsJ-like methyltransferase) and a terpene synthase (polyprenyl synthetase). Notably, *L. ferriphilum* DSM 14647 lacked a series of SecA proteins in the cell membrane of the bacterial Sec or Type II secretion pathway, which led to the obstruction of transmembrane protein synthesis, thereby affecting the transport of terpenoid-precursor compounds inside and outside the cells [62]. *L. ferriphilum* Sp-Cl lacked a long list of genes related to energy transfer and glycosyl transfer, but the mechanism of its impact on terpene-precursor synthesis remained unclear. Therefore, this study identified that most strains of *L. ferriphilum* possess gene clusters for synthesizing terpene precursors. Among them, the most crucial one was Prenyltransferase, which was directly related to polyprenyl synthetase, and may ultimately synthesize C15 sesquiterpene-precursor compounds.

**Figure 6:**
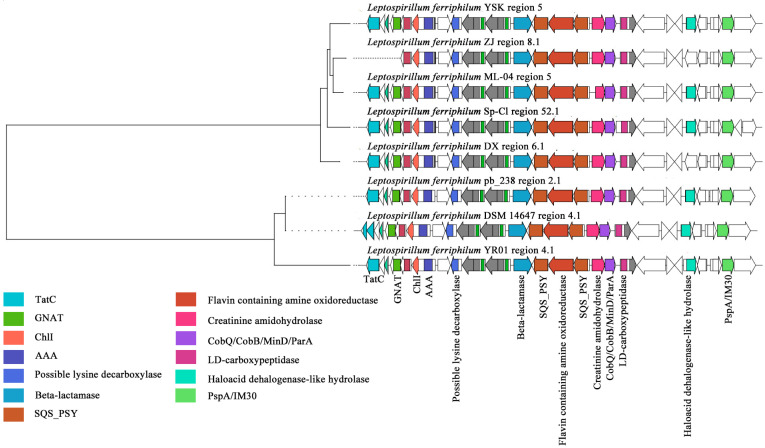
Clustering phylogenetic tree of synthetic gene cluster of *L. ferriphilum* carotenoids.

Within the GCF0030 gene cluster family, the compounds exhibiting the highest predicted similarity to the secondary metabolite BGCs of this family were carotenoids (Figure 6). The BGC contains essential domains associated with carotenoid biosynthesis, notably two squalene/phytoene synthases (SQS-PSYs). Phytoene synthase, encoded by the psy gene, is the initial synthase in the carotenoid biosynthetic pathway. It catalyzes the condensation of two molecules of geranylgeranyl pyrophosphate (GGPP) to form phytoene [63]. Crucially, SQS-PSY serves as the rate-limiting enzyme in the carotenoid metabolic pathway, exhibiting the slowest catalytic reaction rate within the entire pathway and thus playing a pivotal role in regulating the overall biosynthetic process [64]. Additionally, SQS-PSY can participate in squalene biosynthesis, catalyzing the formation of squalene from FPP [65]. In this study, gene cluster analysis revealed that most strains of *L. ferriphilum* possess a complete carotenoid synthesis gene cluster. Furthermore, the SQS-PSY (Prenyltransferase) gene, essential for carotenoid synthesis, potentially facilitates the production of C_30_ or C_40_ carotenoids. Notably, Sec-independent protein translocase protein (TatC) was absent in *L. ferriphilum* ZJ and DSM 14647. The absence of TatC may indirectly affect the enzymes or metabolic pathways related to terpenoid synthesis by interfering with the transport of twin-arginine signal peptide proteins [65]. In addition, *L. ferriphilum* ZJ also lacked the acetyltransferase (GNAT) family. The members of the GNAT family exhibited a certain degree of redundancy, and the absence of some GNATs may be compensated for by other GNATs [66].

**Figure 7:**
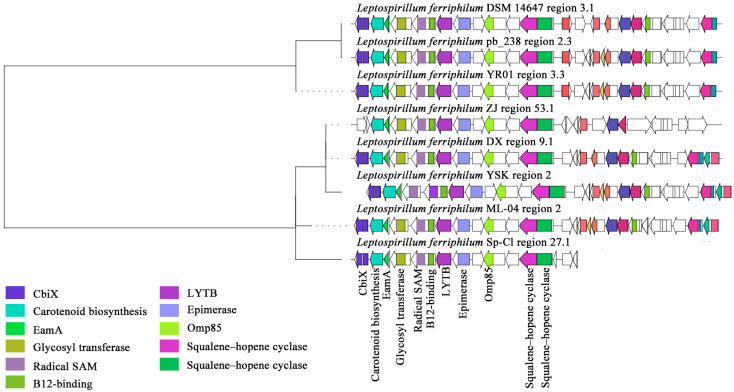
Clustering phylogenetic tree of synthetic gene cluster of *L. ferriphilum* terpenoids.

Within the GCF0029 gene cluster family of *L. ferriphilum*, the sm-BGCs were predicted to exhibit the highest similarity to hopanoids, compounds known for conferring stress tolerance (Figure 7). Especially, analysis also identified the presence of domains associated with carotenoid biosynthesis within this cluster. The detailed predicted terpene-class products for this family are hopanoid-type compounds: hopanoid and polypodane. These metabolites enhance the resistance of acidophilic bacteria to environmental stress by modulating membrane stability, permeability, and fluidity [67]. Key functional domains identified within the cluster include carotenoid biosynthesis protein, cobaltochelatase subunit CbiX, EamA-like transporter family protein, glycosyl transferase family protein, B12-binding domain, radical SAM superfamily enzyme, 4-hydroxy-3-methylbut-2-enyl diphosphate reductase (LytB/IspH), NAD-dependent epimerase/dehydratase, outer membrane protein assembly factor (Omp85), and squalene–hopene cyclase [68]. The ISXO2-like transposase and associated enzymes or genes were present in *L. ferriphilum* YR01, *L. ferriphilum* pb_238, and *L. ferriphilum* DSM 14647, but absent in other strains. The ISXO2-like transposase is a component of a transposon (IS element) belonging to the transposase family with a DDE domain [69]. This type of transposon can regulate gene expression or induce genomic variations through insertion and mobilization within the genome. The absence of the ISXO2-like transposase may lead to decreased transposon activity, potentially reducing dynamic genome recombination and indirectly affecting the expression or stability of certain key genes. This study found that most *L. ferriphilum* strains have complete terpene synthesis gene clusters, with squalene–hopene cyclase (Type 2 terpene synthase) being particularly significant. This enzyme is directly involved in synthesizing hopanoid or polypodane, potentially leading to the production of C_30_ triterpene compounds.

The sequence alignment of secondary metabolite synthesis gene clusters revealed that *L. ferriphilum* can potentially synthesize ectoine, choline substances, AHL, terpene precursors, carotenoids, and hopanoids. Notably, *L. ferriphilum* YR01 uniquely possessed complete BGCs for synthesizing these secondary metabolites, in contrast to the other seven strains examined. This finding suggests that YR01 may exhibit enhanced capabilities for secondary metabolite synthesis and increased adaptability to highly acidic, metal-rich environments.

### 3.3. Identification of Key Secondary Metabolites in Untargeted (LC-MS) Metabolomics

**Figure 8:**
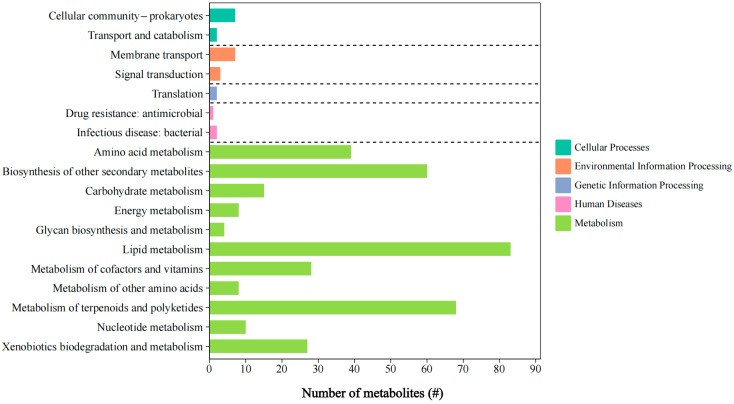
Functional annotations of metabolites produced by *L. ferriphilum* YR01.

Analysis of raw metabolomic data from the selected *L. ferriphilum* YR01 strain led to the curation of 707 metabolites. Tandem mass spectrometry (MS/MS) was performed on characteristic ions (primarily [M + H] in the positive mode and [M − H]^+−^ in the negative mode) of metabolites following spectral feature annotation. To further investigate the role of key metabolites in *L. ferriphilum*, the metabolites detected in *L. ferriphilum* YR01 were annotated, classified, and subjected to pathway enrichment analysis using the KEGG database [70]. The KEGG pathway enrichment results for these metabolites are shown in Figure 8. Only 374 metabolites were successfully annotated with functional information, while 48.8% metabolites remained functionally uncharacterized. This indicated a substantial reservoir of unexplored compounds within *L. ferriphilum* YR01, presenting significant research potential. All predicted metabolites were categorized into five major KEGG functional classes: Metabolism, Genetic Information Processing, Environmental Information Processing, Cellular Processes, and Human Diseases. Within the Metabolism class, Lipid Metabolism, Metabolism of Terpenoids and Polyketides, and Biosynthesis of Other Secondary Metabolites constituted the top three most abundant subcategories. This suggested that key metabolites in the acidophile *L. ferriphilum* YR01 predominantly originate from these three subclasses, with critical secondary metabolites primarily comprising terpenoids and polyketides.

Based on KEGG annotation across all metabolomic datasets, a total of 183 secondary metabolites were annotated in *L. ferriphilum* YR01. In conjunction with the genomic research results, this study focuses on NRPS and Terpene secondary metabolites. The key secondary metabolites are shown in Table 2. Phosphatidycholine (PC) is the major membrane-forming phospholipid in eukaryotes, but it has been found in only a limited number of prokaryotes [71]. This study demonstrated the capacity of *L. ferriphilum* YR01 to synthesize multiple PCs when cultured at pH 2.0. Untargeted metabolomic analysis detected eight distinct PCs with *m*/*z* values ranging from 522.3 to 858.6, corresponding to molecular formulas from C24 to C48. There were multiple key genes potentially associated with PC synthesis in the NRPS-type BGC potentially involved in PC synthesis, with SAM methyltransferase likely playing a critical role [72]. This study suggests that PC biosynthesis in *L. ferriphilum* YR01 primarily occurs via the phosphatidylethanolamine methylation pathway, reliant on phospholipid N-methyltransferase (PMT). In this process, phosphatidylethanolamine (PE) undergoes three methylation steps, using SAM as the methyl donor, to produce PC. This study, drawing on genomic and metabolomic evidence, suggests that in addition to *L. ferriphilum* YR01, several other *L. ferriphilum* strains, including *L. ferriphilum* pb_238, ML-04, DX, Sp-Cl, and ZJ, possess a complete phosphatidylethanolamine methylation pathway. Four QS-related molecules, belonging to three distinct QS systems, were predicted among all metabolites. Crucially, enrichment analysis revealed that *L. ferriphilum* YR01 produced signal molecules associated with AHL-type, DSF-type, and autoinducer-based QS systems. This finding contrasts with previous assumptions by suggesting that only a DSF-type system was present [9,73]. The three distinct QS systems and QS-related molecules include the following: (1) AHL-type QS signal molecule: *N*-(3-Hydroxy-*cis*-7-tetradecenoyl) homoserine lactone (*m*/*z* 695.4); AHL ligand LasR inhibitor: *N*-3-Hydroxyoctanoyl-l-homoserine lactone (*m*/*z* 485.2); (2) DSF-type QS signal molecule: *cis*-11-Methyl-2-dodecenoic acid (*m*/*z* 213.1); (3) autoinducer for a Two-Component System (also a QS signal molecule): CAI-1 (*m*/*z* 446.4), a molecule originally identified in *Vibrio cholerae* [18].

The untargeted metabolomics analysis of *L. ferriphilum* YR01 revealed its capacity to synthesize diverse terpenoids and their precursors, including three types of carotenoids. Two key intermediates in the biosynthesis of C_15_ sesquiterpenoids were identified, i.e., Germacrene A acid (*m*/*z* 279.1, C_15_H_22_O_2_) and Costunolide (*m*/*z* 523.3, C_15_H_20_O_2_). Germacrene A is produced through the cyclization of FPP, catalyzed by Germacrene A synthase [74,75]. Germacrene A then undergoes multiple oxidation reactions, mediated by Germacrene A oxidase, to form Germacrene acid. Costunolide is subsequently synthesized from Germacrene acid through hydroxylation at the C6 position and subsequent lactone cyclization, catalyzed by Costunolide synthase [76]. Based on the genomic and metabolomic evidence, it is speculated that, in addition to *L. ferriphilum* YR01, *L. ferriphilum* pb_238, ML-04, YSK, DX, and ZJ may also possess the complete biosynthetic pathway for the key intermediates in sesquiterpenoid production.

Untargeted metabolomics studies indicate that *L. ferriphilum* YR01 synthesizes a triterpene, 2,3-Bis-O-(geranylgeranyl)-sn-glycero-1-phospho-l-serine (*m*/*z* 802.5, C_46_H_78_NO_8_P), and three carotenoids: Antheraxanthin (a tetraterpene C_40_ carotenoid, *m*/*z* 583.4, C_40_H_56_O_3_), Neurosporaxanthin (a tetraterpene C_35_ carotenoid, *m*/*z* 543.3, C_35_H_46_O_2_), and 4,4′-Diaponeurosporene (a triterpene C_30_ carotenoid, *m*/*z* 447.3, C_30_H_42_). In the extremely acidic and high-iron environment of *L. ferriphilum*, carotenoids played a crucial role in scavenging reactive oxygen species (ROS) and protecting cellular components, particularly membrane lipids, from oxidative damage [77]. The biosynthesis of Antheraxanthin is catalyzed by the enzyme zeaxanthin epoxidase (ZEP), which converts zeaxanthin into Antheraxanthin [78]. Antheraxanthin can then be further converted into other carotenoids [79]. Neoxanthin is generated from Antheraxanthin through the action of violaxanthin de-epoxidase and the carotenoid synthase NSY [80]. The biosynthesis of 4,4′-Diaponeurosporene is catalyzed by two enzymes: dehydrosqualene synthase (CrtM) and dehydrosqualene desaturase (CrtN). CrtM converts two molecules of farnesyl pyrophosphate (FPP) into 4,4′-diapophytoene, which is then converted into 4,4′-diaponeurosporene by CrtN through a dehydrogenation reaction [81]. Genomic and metabolomic studies suggest that, in addition to *L. ferriphilum* YR01, other strains such as *L. ferriphilum* pb_238, ML-04, YSK, and Sp-Cl may also possess multiple carotenoid biosynthetic pathways. Sequence alignment and evolutionary analysis indicated that hopanoid-associated genes frequently co-localize with carotenoid biosynthesis genes within BGCs. This genomic co-localization suggested a close functional linkage between the biosynthesis of these two metabolite classes, potentially involving shared biosynthetic pathways or co-regulation [82].

### 3.4. Identification of Key Secondary Metabolites Using HPLC-MS

**Figure 9:**
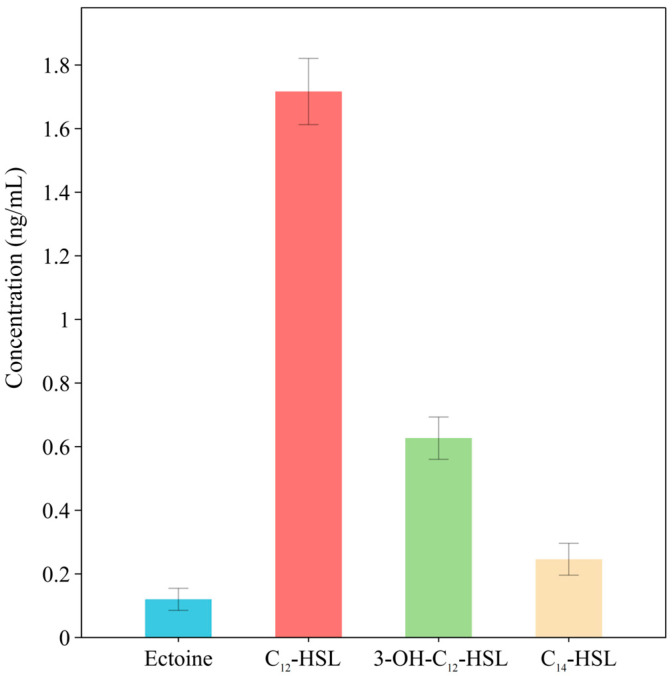
SMs detected in *L. ferriphilum* YR01 using targeted metabolomics.

Based on the prediction of secondary metabolite BGCs, this study identified the presence of the complete ectoine biosynthesis genes *ectA*, *ectB*, *ectC*, and *ectD* in *L. ferriphilum* YR01. However, ectoine was not detected in untargeted analyses, potentially due to the lower sensitivity of untargeted metabolomics or its predominant production during early growth stages, with reduced metabolic output during mid-to-late phases [12]. Consequently, the detection of ectoine secreted during early-to-mid growth phases under cultivation with FeSO_4_·7H_2_O as the energy source was performed. Using HPLC-MS, trace amounts of ectoine (0.10 ng/mL) were successfully detected (Figure 9). The targeted detection results validated the BGC predictions, further confirming that *L. ferriphilum* YR01 had the genetic capability for ectoine biosynthesis and could secrete this natural product under extremely acidic conditions (pH 2.0). Targeted detection identified ectoine ([M + H]^+^ = 143) as an osmoprotectant-compatible solute. This marked the first detection of *L. ferriphilum* YR01 synthesizing ectoine in a high heavy metal concentration environment, challenging the prior belief that its synthesis was restricted to high-salt conditions [68].

Similarly, based on the prediction of AHL synthase domains within NRPS-like BGCs, we performed the targeted quantification of AHL signaling molecules. *L. ferriphilum* YR01 was cultivated using FeSO_4_·7H_2_O as the sole energy source. AHLs secreted by the strain were identified via HPLC-MS. Three AHLs were confirmed under pure culture conditions, i.e., C_14_-HSL (*N*-tetradecanoyl-l-homoserine lactone), C_12_-HSL (*N*-dodecanoyl-l-homoserine lactone), and C_12_-OH-HSL (*N*-(3-hydroxydodecanoyl)-l-homoserine lactone), exhibiting [M + H]^+^ values of *m*/*z* 312.2, 284.3, and 300.3, respectively. The MS/MS fragmentation patterns of these three AHLs matched those of authentic standards. The relative abundance of each AHL in the samples was determined using peak area normalization. Results are presented in Figure 9: when grown with 60 g/L FeSO_4_·7H_2_O, the highest production level was observed for C_12_-HSL (1.8 ng/mL), followed by C_12_-OH-HSL (0.61 ng/mL) and C_14_-HSL (0.25 ng/mL).

Untargeted metabolomic analysis revealed that *L. ferriphilum* YR01 could synthesize four distinct QS signal molecules: *N*-Heptanoylhomoserine lactone, *N*-3-Hydroxyoctanoyl-l-homoserine lactone, *N*-(3-Hydroxy-7-*cis*-tetradecenoyl) homoserine lactone, and *cis*-11-Methyl-2-dodecenoic acid. Subsequent identification further identified three specific AHL-type QS signals (C_14_-HSL, C_12_-HSL, C_12_-OH-HSL) in this strain. This represented the first direct detection of AHL-based QS in *L. ferriphilum*, challenging the previous assumption that this species solely produces DSF-type QS signaling molecules. Previous studies reported that DSF-type quorum sensing was the only known signaling mechanism in *Leptospirillum* spp., which typically functions as a density-dependent inhibitory signal limiting excessive cell growth or biofilm formation. In contrast, the additional QS molecules identified in this study, including AHLs and CAI-1, are often associated with positive regulatory effects on cooperative metabolism and bioleaching efficiency. This finding suggests that *L. ferriphilum* may employ a combination of inhibitory (DSF) and stimulatory (AHL, CAI-1) signaling systems to fine-tune population dynamics and enhance adaptation under extreme AMD conditions. Integrating the genomic and metabolomic evidence, we speculate that the ability to synthesize AHL QS signals may be conserved across other *L. ferriphilum* strains, such as *L. ferriphilum* pb_238 and ML-04.

The identified metabolites, including ectoine, phosphocholine, and carotenoids, are closely associated with stress adaptation in extremely acidic and metal-rich environments. Ectoine act as compatible solutes that stabilize proteins and membranes under osmotic and acid stress, mitigating oxidative damage caused by heavy metals. These metabolites collectively contribute to the enhanced environmental adaptation of *L. ferriphilum* in AMD systems.

## 4. Conclusions

The acidophile *L. ferriphilum* YR01 was isolated from the AMD at the Zijinshan copper mine, China. Genomic analysis revealed that YR01 possesses the largest gene repertoire (2623 genes) among the eight sequenced *L. ferriphilum* strains. The sequence analysis of sm-BGCs demonstrated the capacity of *L. ferriphilum* (harboring 39 BGCs, with 60% showing <50% similarity to known clusters) to synthesize conserved clusters for ectoine, choline, carotenoids, terpenoids, and terpene precursors. Notably, YR01 harbors complete BGCs for all five SM classes. Key NRPS modules for AHL synthesis were identified, and this study demonstrated for the first time in *L. ferriphilum* the direct production of ectoine and multiple AHLs via HPLC-MS. Untargeted metabolomics revealed YR01’s production of 18 diverse specialized metabolites, potentially contributing to environmental adaptation, including phosphocholine, carotenoids (e.g., anteraxanthin), CAI-1, and multiple AHLs. Targeted detection confirmed YR01’s production of ectoine (0.10 ng/mL) and several AHLs (C_14_-HSL, C_12_-HSL, C_12_-OH-HSL). Collectively, this study quantitatively establishes YR01 as a metabolically versatile acidophile with vast unexplored biosynthetic potential. It also highlights the biotechnological potential of these secondary metabolites for biomining applications and for engineering microorganisms resilient to extreme conditions. Furthermore, the multi-omics pipeline employed here can be extended to investigate other acidophilic communities in AMD systems, offering a scalable framework for functional exploration of microbial secondary metabolism. These findings fundamentally advance our understanding of how *L. ferriphilum* survives and functions in harsh biomining niches. The identified sm-BGCs offer biotechnological potential, including ectoine biosynthesis, biomining efficiency enhancement via QS manipulation, and the discovery of stress-adapted enzymes. However, the current study does not provide direct experimental evidence to confirm these effects. Future work, including transcriptomic analyses or targeted mutagenesis, will be necessary to elucidate how these gene cluster arrangements impact metabolite production and adaptive responses under AMD conditions. At the same time, future work should also explore the heterologous expression of uncharacterized BGCs (60% with <50% similarity to known clusters) to develop new metabolites adapted to extreme environments. In addition, future heterologous expression of these uncharacterized BGCs could uncover novel bioactive metabolites with potential industrial relevance.

## Figures and Tables

**Table 2:** Untargeted metabolomics analysis of *L. ferriphilum* YR01.

BGC Type	Product Type	Name	*m*/*z*	Formula	Relative Abundance
NRPS	Phosphocholine	1,2-dioleoyl-sn-glycero-3-phosphatidylcholine	830.5	C_44_H_84_NO_8_P	35.78
NRPS	Phosphocholine	1-Palmitoyl-sn-glycero-3-phosphocholine	540.3	C_24_H_50_NO_7_P	275.55
NRPS	Phosphocholine	PC(18:0/0:0)	522.3	C_26_H_54_NO_7_P	132.53
NRPS	Phosphocholine	PC(o-18:0/20:4(8Z,11Z,14Z,17Z))	816.5	C_46_H_86_NO_7_P	10,319.90
NRPS	Phosphocholine	PC(o-16:0/22:6(4Z,7Z,10Z,13Z,16Z,19Z))	790.5	C_46_H_82_NO_7_P	58,351.83
NRPS	Phosphocholine	PC(18:1(11Z)/20:5(5Z,8Z,11Z,14Z,17Z))	850.5	C_46_H_80_NO_8_P	5044.68
NRPS	Phosphocholine	PC(22:2(13Z,16Z)/P-18:1(9Z))	858.6	C_48_H_90_NO_7_P	12,887.84
NRPS	Phosphocholine	PC(14:0/22:1(13Z))	832.6	C_44_H_86_NO_8_P	14,430.83
NRPS	AHL-QQ	*N*-3-Hydroxyoctanoyl-l-homoserine lactone	485.2	C_12_H_21_NO_4_	49,069.18
NRPS	AHL	*N-*(3-Hydroxy-7-*cis*-tetradecenoyl)homoserine lactone	695.4	C_18_H_31_NO_4_	220.42
NRPS	DSF	*cis*-11-Methyl-2-dodecenoic acid	213.1	C_13_H_24_O_2_	342.77
NRPS	AI-1	CAI-1	446.4	C_13_H_26_O_2_	151.09
Terpene	Triterpene	2,3-Bis-O-(geranylgeranyl)-sn-glycero-1-phospho-l-serine	802.5	C_46_H_78_NO_8_P	36,021.37
Terpene	C_40_ carotenoid	Antheraxanthin	583.4	C_40_H_56_O_3_	496.95
Terpene	C_35_ carotenoid	Neurosporaxanthin	543.3	C_35_H_46_O_2_	720.62
Terpene	C_30_ carotenoid	4,4′-Diaponeurosporene	447.3	C_30_H_42_	43.37
Terpene	Sesquiterpene precursor	Germacrene A acid	279.1	C_15_H_22_O_2_	9459.19
Terpene	Sesquiterpene precursor	Costunolide	523.3	C_15_H_20_O_2_	25.94

## Data Availability

The data presented in this study are openly available in National Center for Biotechnology Information at https://www.ncbi.nlm.nih.gov/ (accessed on 1 April 2025), reference number PRJNA1281278.

## Abbreviations

The following abbreviations are used in this manuscript:

AMD	Acid Mine Drainage
BGC	Biosynthetic Gene Cluster
SM	Secondary Metabolites
NRPS	Nonribosomal Peptide Synthetase
CAI-1	Cholera Autoinducer-1
AHL	*N*-acyl-homoserine Lactone
LC-MS	Liquid Chromatography–Mass Spectrometry
HPLC-MS	High-Performance Liquid Chromatography–Mass Spectrometry
DSF	Diffusible Signal Factor
QS	Quorum Sensing
PKS	Polyketide Synthase
MIC	Minimal Inhibitory Concentration
RiPPs	Ribosomally Synthesized and Post-translationally modified Peptides
NCBI	National Center for Biotechnology Information
OrthoANI	Orthologous Average Nucleotide Identity
BPGA	Bacterial Pan Genome Analysis
BiG-SCAPE	Biosynthetic Gene Cluster Similarity Analysis and Prediction Engine
QC	Quality Control
GCFs	Gene Cluster Families
TetR	Tet Repressor
PhyH	Phytanoyl-CoA Dioxygenase
DABA	2,4-Diaminobutyric Acid
ADABA	*N*-γ-Acetyl-2,4-Diaminobutyric Acid
SAM	S-adenosylmethionine
SDR	Short-Chain Dehydrogenase/Reductase
ArsR	Arsenical Pump Membrane Protein
HMA	Heavy-metal-associated
ACP	Acyl Carrier Protein
VOC	Volatile Organic Compound
FPP	Farnesyl Pyrophosphate
SQS-PSY	Squalene/phytoene Synthase
GGPP	Geranylgeranyl Pyrophosphate
GNAT	GCN5-related *N*-acetyltransferases
Omp85	Outer Membrane Protein 85
PC	Phosphatidycholine
PMT	Phospholipid *N*-methyltransferase
PE	Phosphatidylethanolamine
ROS	Reactive Oxygen Species
ZEP	Zeaxanthin Epoxidase
CrtM	Dehydrosqualene Synthase
CrtN	Dehydrosqualene Desaturase
C_14_-HSL	*N*-tetradecanoyl-l-homoserine Lactone
C_12_-HSL	*N*-dodecanoyl-l-homoserine Lactone
C_12_-OH-HSL	*N*-(3-hydroxydodecanoyl)-l-homoserine Lactone
